# Thromboembolic events and haematological diseases: a case of stroke as clinical onset of a paroxysmal nocturnal haemoglobinuria

**DOI:** 10.1186/1477-9560-2-10

**Published:** 2004-11-11

**Authors:** Gianluca Granata, Tiziana Izzo, Pierpaolo Di Micco, Barbara Bonamassa, Giampiero Castaldo, Vito Giuseppe Viggiano, Ugo Picillo, Giuseppe Castaldo, Alferio Niglio

**Affiliations:** 1 Internal Medicine of Second University of Naples, Naples, Italy; 2 University of Molise, Aesernia, Italy

**Keywords:** paroxysmal nocturnal haemoglobinuria, stroke, thromboembolic events, haematological disease

## Abstract

Some haematological diseases are associated to an increased risk of thromboembolic events. We report a case of paroxysmal nocturnal haemoglobinuria (PNH) in which a cerebrovascular event represented the first clinical manifestation of disease. PNH is associated to thromboembolic events, generally of venous districts often involving unusual locations such as mesenteric vessels, sagittal veins, inferior vena cava and renal veins.

To our knowledge arterial thrombotic episodes are rare and the involvement of arterial cerebral vessels is exceptional. Then, our case points out the importance of investigating about haematological disorders in all patients presenting with a stroke, in which the common predisposing conditions are excluded.

## Background

Paroxysmal nocturnal haemoglobinuria (PNH) is an acquired clonal disorder of haematopoietic stem cells clinically characterized by acute intravascular haemolytic crisis, in particular nocturnal, often overlapped to chronic haemolysis, and by thrombotic events and bone marrow failure. It is associated with a somatic mutation in the phosphatidylinositol glycan complementation class A (PIG-A) gene, mapped to the X chromosome; the subsequent deficiency of glycosylphosphatidylinositol (GPI) and of GPI-anchored molecules, as the decay accelerating factor (DAF or CD55) and the membrane inhibitor of reactive lysis (MIRL or CD59), causes an increased susceptibility to complement-mediated lysis of erythrocytes, leukocytes and platelets [1].

The association between PNH and thromboembolic accidents, generally manifesting as thrombotic events of venous vessels sometimes complicated by pulmonary embolism, is well established. Arterial thrombotic episodes, particularly of cerebral vessels are enough rare [2].

We report a case of PNH presenting with thromboembolic events, both venous (proximal deep venous thrombosis of lower limbs) and arterial (stroke).

## Case history

### Clinical summary

A 56-year-old woman, with history of peptic ulcer and family history for cerebrovascular disease was referred to our Division of Internal Medicine with asthenia and generalized discomfort. She reported a cerebrovascular accident manifesting as a right brachial and crural hyposthenia ten month ago, almost completely receded at observation time; she also referred recurrent episodes of proximal deep venous thrombosis (DVT) of lower limbs in the last seven months.

#### Pathological findings

In order to identify any hypercoagulable state (i.e. inherited or acquired thrombophilia), in view of her personal and familiar history, we tested prothrombin time, as INR, activated partial thromboplastin time, as ratio, fibrinogen, protein C and S, antithrombin III, activated protein C resistance, anti-cardiolipin antibodies IgG and IgM, lupus anticoagulant, plasminogen activator inhibitor type 1, d-dimer, gene polimorphism of clotting factor II and V, gene polimorphism C9774T and G3775A of apolipoprotein B and gene polimorphism C3932T and C4070T of apolipoprotein E resulted all in normal range; while gene polimorphism of tetrahydrofolate reductase and angiotensin converting enzyme revealed heterozigosity for both. Subsequently, homocysteinemia test revealed mild hyperhomocysteinemia. All thrombophilic tests are summarised in table 1.

**Table 1:** Thrombophilic tests

**Thrombophilic tests **(units of measurement)	**Results**	**Normal range**
Protein C (antigen) (%)	99%	60–125
Protein S (antigen) (%)	102%	60–125
Antithrombin (activity) (%)	105%	80–120
Activated protein C resistance (Bertina)	0,90	>0,77
Anti-cardiolipin antibodies IgG (U/GPL)	4	<7
Anti-cardiolipin antibodies IgM (U/MPL)	2	<4
Lupus anticoagulant	absent	absent
Plasminogen activator inhibitor type 1 (ng/dl)	30	4–44
PTHRA20210 gene polimorphism	wild type	wild type
Factor V Leiden gene polimorphism	wild type	wild type
Apolipoprotein B gene polimorphism C9774T and G3775A	wild type	wild type
Apolipoprotein E gene polimorphism C3932T and C4070T	wild type	wild type
Methylene-tetrahydrofolate C677T gene polimorphism	heterozigosity	wild type
Angiotensin converting enzyme deletion gene polimorphism	insertion/deletion	insertion/insertion
Homocysteinemia (μM)	22	5–15
Prothrombin time (INR)	0.95	0.8–1.2
Activated partial thromboplastin time (ratio)	0.92	0.8–1.2
Fibrinogen (mg/dl)	305	220–400
D-dimer (ug/l)	188	0–198

**Figure 1:**
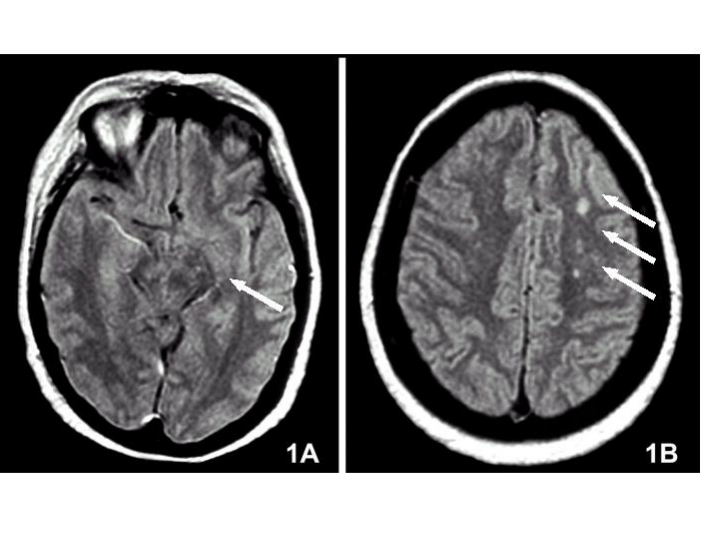
Magnetic resonance imaging scan showing multiple ischemic lesions in left cerebral peduncle (1A) and semioval centres (1B).

A magnetic resonance imaging scan showed little and multiple ischemic lesions in particular in left cerebral peduncle (fig 1A), semioval centres (fig 1B), left pons and midbrain. Moreover, a vascular ultrasound examination ruled out the presence of significant stenosis of arterial cerebral vessels and confirmed proximal DVT and post-thrombotic syndrome of lower limbs.

Other available data showed: red blood cells 2.470.000/mm^3^, hemoglobin 7.9 g/dl, hematocrit 25%, mean corpuscular volume 99,6 fl, mean corpuscular hemoglobin 32 pg, mean corpuscular hemoglobin concentration 32 gr/dl, white blood cells 4,040/mm^3^, platelets 93.000/mm^3^, reticulocytes 5,4%, serum iron 76 μg/dl, erytro-sedimentation rate 1° hour 40 mm, lactate dehydrogenase 944 UI/l, total bilirubin 0,72 mg/dl, indirect bilirubin 0,36 mg/dl, and presence of hemoglobinuria. Coombs' test, cold agglutinins, antinuclear antibodies, anti-extractable nuclear antigens antibodies, anti-mithocondrial antibodies, anti-smooth muscle antibodies were negative. Laboratory data and their range are summarised in table 2.

**Table 2:** Other laboratory findings

**Laboratory data **(units of measurement)	**Results**	**Normal range**
Erytro-sedimentation rate 1° hour (mm)	40	<10
lactate dehydrogenase (UI/l)	944	100–190
total bilirubin (mg/dl)	0,72	0–1
indirect bilirubin (mg/dl)	0,36	0–0,5
antinuclear antibodies	absent	absent
anti-extractable nuclear antigens antibodies	absent	absent
anti-mithocondrial antibodies	absent	absent
anti-smooth muscle antibodies	absent	absent

An abdominal ultrasonography excluded a hypersplenism and/or Kasabath-Merritt syndrome.

A peripheral blood smear did not show any finding suggestive for haematological disorders. A bone marrow biopsy showed a slight hyperplasia of erythrocytic bone marrow cell line.

These laboratory and morphological findings suggested a non-immune haemolytic anemia. In particular, due to the exclusion of other non-immune haemolityc disorders by means of age and clinical history together with the presence of hemoglobinuria and pancytopenia, we hypothesized paroxysmal nocturnal hemoglobinuria. This diagnosis was confirmed by an immunophenotypic profile of peripheral blood cells, showing a 15% of deficient CD59 erythrocytes, and by the presence of hemosiderinuria. Haematological findings are summarised in table 3.

**Table 3:** Haematological data

**Laboratory data **(units of measurement)	**Results**	**Normal range**
red blood cells (cells/mm^3^)	2.470.000	4.200.000 – 5.400.000
hemoglobin (g/dl)	7,9	12–16
hematocrit (%)	25	37–45
mean corpuscolar volume (fl)	99,6	81–99
mean corpuscolar hemoglobin (pg)	32	27–31
mean corpuscolar hemoglobin concentration (g/dl)	32	32–36
white blood cells (cells/mm^3^)	4.040	4.800 – 10.800
Platelets (cells/mm^3^)	93.000	130.000 – 400.000
Reticulocytes (%)	5,4	<2
haemoblobinuria	traces	absent
Hemosiderinuria	present	absent
Coombs'test	negative	negative
cold agglutinins	negative	negative
Peripheral blood smear	Normal	
Bone marrow biopsy	slight hyperplasia of erythrocytic cell line	
Immunophenotypic profile of peripheral blood cells	15% of deficient CD59 erythrocytes	

During her hospitalization two haemotrasfusions were necessary in occasion of two concurrent haemolytic crises. Following dismission, in order to prevent further thromboembolic events, the patient began oral anticoagulation therapy with warfarin according with INR value in range of 2–2,5. Moreover she was treated with B12 vitamin and folate supplementation.

## Discussion

The association between haematological diseases and thromboembolic events is well established. In particular high thrombotic risk is recognized in patients with essential thrombocythemia, polycythemia vera, PNH and drepanocytosis [3]. PNH is associated to venous thrombosis in approximately one third of cases. The most frequently reported locations are unusual such as mesenteric vessels, sagittal veins, inferior vena cava and renal veins. When thrombosis occurs in the pre-hepatic or hepatic veins, the patient develops a Budd-Chiari syndrome [4]. Arterial thrombosis is rare, even if few cases of cerebral arterial thrombosis [5] and acute myocardial infarction [6] are described in the literature.

The mechanism whereby PNH causes an hypercoagulable state is not clear. PNH platelets lack the GPI-linked proteins CD55 and CD59, and respond to the deposition of terminal complement components by vesiculations of portions of their plasma membrane, resulting an increased procoagulant property. PNH cells also lack the receptor of the GPI-linked urokinase plasminogen activator, which may result in impaired fibrinolysis [4]. Also an increase of membrane-derived procoagulant microparticles (phosphatidylserin) stemming from the platelets of PNH patients has been described [3].

In our case, thrombotic events represented the clinical onset of PNH and involved both venous (DVT) and arterial (stroke) vessels. Neurological manifestations in PNH patients are generally due to cerebral venous thrombosis [7,8], even if a few cases of cerebral arterial episodes, involving large vessels, are described. However, usually cerebral ischaemia in PNH did not occur as presenting sign of the disease nor affect small and middle cerebrovascular arteries [5]. In our patient the relationship between PNH and thrombotic events is strongly suggested, especially after excluding inherited or acquired thrombophilia and atherosclerotic risk factors. Heterozigosities for gene polimorphism of tetrahydrofolate reductase and angiotensin converting enzyme, detected in our patient, are not associated to an increased risk of stroke, while acquired or inherited hyperhomocysteinemia may be involved [9-11].

Also haemotological findings agree with PNH diagnosis because of the association of thrombosis, anemia and thrombocytopenia. We excluded further causes of non-immune haemolityc anemia (i.e. spherocytosis, enzymatic disorders, microangiopathic anemia) and thrombocytopenia (i.e. disseminated intravascular coagulation, haematological malignancies, systemic erythematosus lupus, primary or secondary antiphospholipid syndrome, hypersplenism).

In conclusion, PNH is associated to thromboembolic events, especially in the venous district and should be considered as a possible cause of an hypercoagulable state, in particular when unusual vascular locations are involved. Our case indicates the possibility of arterial thrombotic episodes in a patient with PNH and suggests a thorough evaluation of any haematological disorders in patients presenting with stroke or myocardial infarction, especially in the absence of atherosclerosis risk factors and/or a thrombophilic state.
